# How to Perform a Microfluidic Cultivation Experiment—A Guideline to Success

**DOI:** 10.3390/bios11120485

**Published:** 2021-11-29

**Authors:** Sarah Täuber, Julian Schmitz, Luisa Blöbaum, Niklas Fante, Heiko Steinhoff, Alexander Grünberger

**Affiliations:** 1 Multiscale Bioengineering, Faculty of Technology, Bielefeld University, Universitätsstraße 25, 33615 Bielefeld, Germany; sarah.taeuber@uni-bielefeld.de (S.T.); j.schmitz@uni-bielefeld.de (J.S.); luisa.bloebaum@uni-bielefeld.de (L.B.); niklas.fante@uni-bielefeld.de (N.F.); heiko.steinhoff@uni-bielefeld.de (H.S.); 2 Center for Biotechnology (CeBiTec), Bielefeld University, Universitätsstraße 27, 33615 Bielefeld, Germany

**Keywords:** microfluidics, microfluidic cultivation, single-cell cultivation, microfluidic troubleshooting, live-cell imaging, microfluidic case studies

## Abstract

As a result of the steadily ongoing development of microfluidic cultivation (MC) devices, a plethora of setups is used in biological laboratories for the cultivation and analysis of different organisms. Because of their biocompatibility and ease of fabrication, polydimethylsiloxane (PDMS)-glass-based devices are most prominent. Especially the successful and reproducible cultivation of cells in microfluidic systems, ranging from bacteria over algae and fungi to mammalians, is a fundamental step for further quantitative biological analysis. In combination with live-cell imaging, MC devices allow the cultivation of small cell clusters (or even single cells) under defined environmental conditions and with high spatio-temporal resolution. Yet, most setups in use are custom made and only few standardised setups are available, making trouble-free application and inter-laboratory transfer tricky. Therefore, we provide a guideline to overcome the most frequently occurring challenges during a MC experiment to allow untrained users to learn the application of continuous-flow-based MC devices. By giving a concise overview of the respective workflow, we give the reader a general understanding of the whole procedure and its most common pitfalls. Additionally, we complement the listing of challenges with solutions to overcome these hurdles. On selected case studies, covering successful and reproducible growth of cells in MC devices, we demonstrate detailed solutions to solve occurring challenges as a blueprint for further troubleshooting. Since developer and end-user of MC devices are often different persons, we believe that our guideline will help to enhance a broader applicability of MC in the field of life science and eventually promote the ongoing advancement of MC.

## 1. Introduction

Microfluidic technologies have provided a multitude of tools for manipulating and analysing small volumes of fluid to control and study chemical, biological and physical processes [1]. The miniaturisation of fluidic and optical components in microfluidic devices has been used to study the behaviour of small volumes of fluids in microfluidic channels with a scale of few micrometres to investigate effects that are usually neglected at the macroscopic level. The advantages of this technique include very low Reynolds numbers resulting in strictly laminar flow [2], where mixing is mainly due to molecular diffusion at the interface of two liquids [3], high surface to volume ratio [4], surface tension [4] and low volume of fluids (µL to pL) [5]. Applied microfluidic devices are manufactured by micromachining [6], soft lithography [7], embossing [6], in-situ construction [8,9], injection moulding [10], laser cutting [11], and stereolithography (3D printing) [12].

**Figure 1:**
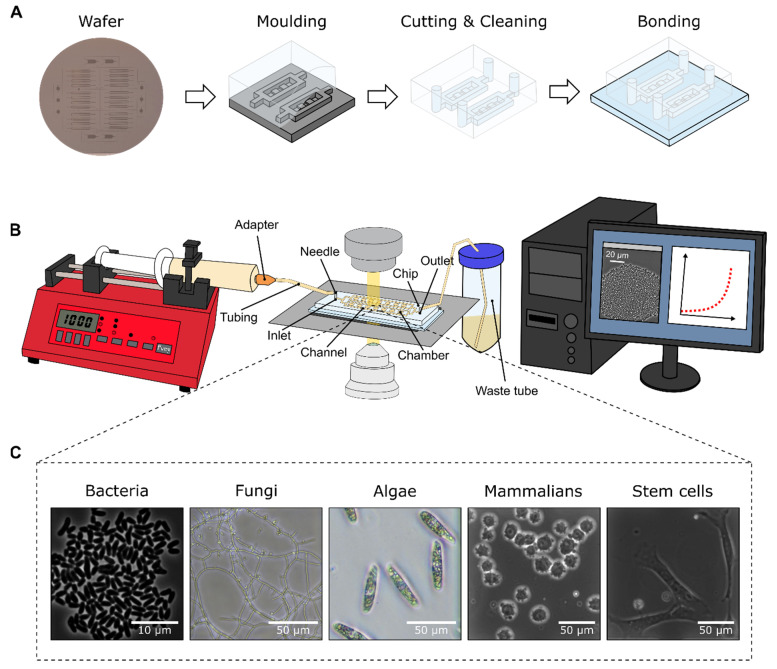
Overview of a microfluidic cultivation (MC) experiment. (**A**) Fabrication of a MC device: moulding a silicon wafer with PDMS, cutting, and cleaning the resulting PDMS chip, bonding of the PDMS chip with a glass substrate. (**B**) Experimental setup with a live-cell imaging microscope for time-lapse microscopy under constant environmental conditions. (**C**) Overview of different organisms, namely bacteria, fungi, algae, mammalian suspension cells as well as adherent human stem cells, that have been analysed using MC so far.

However, soft lithography is the most common method to fabricate microfluidic devices. Here, by casting a mould of a two-component polymer named polydimethylsiloxane (PDMS), a microfluidic chip is generated (Figure 1A). Since production is fast and inexpensive, soft lithography is suitable for rapid prototyping [13]. Additionally, PDMS is a biocompatible and transparent material, thus enabling plentiful biological and biotechnological applications like microfluidic cultivation (MC) and live-cell imaging of different organisms.

In recent years, more and more biologists and bioengineers started to use microfluidic devices for the cultivation and analysis of different organisms, as they can be flexibly designed to fit the requirements of different investigational approaches. MC allows capturing and cultivating small cell clusters or even single cells in microfluidic structures or droplets, ranging from microlitre to picolitre volumes. Droplet microfluidics are not further discussed in this paper, as the focus lies on microfluidic devices that are operated under continuous-flow conditions. This steady perfusion permits the very precise control of cultivation conditions or even reliable environmental changes if requested, so that natural cellular microenvironments or bioprocess conditions can be mimicked [14]. When MC is combined with live-cell imaging, time-lapse microscopy allows the analysis of cellular behaviour in a timely resolved manner (Figure 1B). Because of its miniaturised scale, MC additionally facilitates a high degree of parallelisation and thus increases experimental throughput in comparison to ordinary cultivation attempts.

For the cultivation and analysis of cells in MC devices, different cell trapping approaches such as hydrodynamic [15,16,17], electrical [18], optical [19], or magnetic trapping [20] as well as acoustic methods [21] are available. Due to its plain experimental setup and straightforward fabrication, hydrodynamic trapping in so called cultivation chambers, which range from 3D to 0D, is the most common approach for MC. The required chamber-based devices differ in the spatial degree of freedom for cellular growth and the maximal size of observed microcolonies [14]. In 3D cultivation chambers, the fabrication as well as the seeding procedure is easy, while nutrient gradients can be observed frequently during cultivation and the tracking of cells is difficult as cells do not reside in one focal plane. These chamber structures are often used to study tissues and densely packed cultures starting from a few cells. Most common are 2D cultivation chambers, which allow the cultivation of small monolayered microcolonies [22]. Fabrication and cell seeding is comparable to 3D structures, however, monitoring and analysis of cellular behaviour such as colony growth, cell division and morphology is superior in 2D designs. 1D cultivation chambers, also called mother machines, are best suited for the analysis of cells over multiple generations without spatial restriction of a 2D chamber design, as newly originating cells push out their ancestor cells. These chambers are designed to grow cells in a line so that the parent cell is at the end of the chamber and daughter cells can be examined. This allows long-term culturing of cells over a large number of generations (> 50), as tracking of cells is easy. Thus, cell dynamics can be studied and phylogenetic trees can be constructed. In 0D cultivation chambers only single cells can be trapped and cultivated, which allows cellular analyses of isogenic cells. Therefore, no cellular interaction or cell-to-cell communication take place so that single-cell behaviour of individually cells can be investigated. Long-term studies of cellular behaviour as well as adaptation studies can be performed over a number of generations. For more detailed description of the different chamber designs the reader is referred to Grünberger et al. [14]. Currently, MC is applied for the analyses of cell-to-cell heterogeneity [23], aging and death [23,24,25,26], growth [24,27,28,29], cell cycle monitoring [30], gene expression [31,32,33,34], and metabolic processes [35,36]. Organisms that have been applied for MC analyses are bacteria [37], cyanobacteria [38], eukaryotic cells [39,40], algae [41], fungi [42], and yeast [43] (Figure 1C).

Most of the MC devices are custom-made without any standardisation, which makes their application particularly difficult for beginners in the field of microfluidics. Additionally, self-made setups are often prone to recurring problems along the whole workflow of an experiment. To make MC accessible for microfluidic non-proficient end-users, a problem-oriented troubleshooting guide for the most frequently occurring challenges is provided here. As already indicated, only PDMS-based and chamber-based microfluidic devices are described and discussed. Introducing the field of MC, the conventional experimental workflow will be summarised briefly in its seven consecutive steps: microfluidic design and fabrication, PDMS chip assembly, cell and medium preparation, hardware preparation, device loading, cultivation, and live-cell imaging. For more detailed descriptions of the single steps, the reader is referred to relevant literature in the respective section. Following the workflow, the most prominent challenges in all steps along the MC pipeline are listed and different solutions are given in our in-detail troubleshooting guide. Finally, three case studies which take a closer look at different challenges during MC experiments are presented: establishing MC for a new organism, performing reproducible MC experiments, and performing negative-control experiments. These case studies serve as a blueprint for solving complex challenges concerning the cultivation of cells in microfluidic devices.

## 2. Experimental Workflow

**Figure 2:**
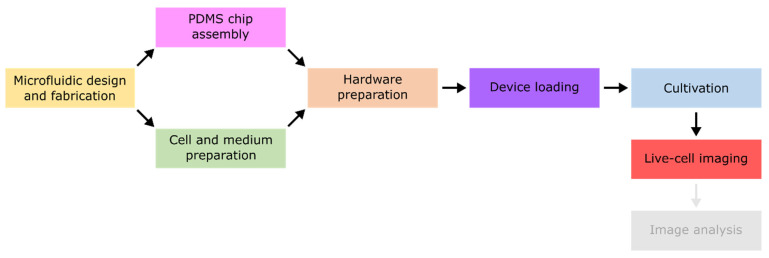
Flow diagram summarising the most important steps during a MC experiment. The experimental workflow is divided into seven consecutive steps: Microfluidic design and fabrication, PDMS chip assembly, cell and medium preparation, hardware preparation, device loading, cultivation, and live-cell imaging. Since the subsequently required image analysis is not a part of this work, it is blurred.

In the following, the general workflow of MC experiments performed in a PDMS-glass-based device (Figure 1B), referred in the following as microfluidic chip, is discussed. To fabricate and operate such a device, there is a strict order in experimental operations, that can be divided into seven consecutive steps (Figure 2). Before the PDMS chip can be fabricated by soft lithography and the MC device can be assembled, a proper microfluidic design needs to be devised and manufactured into a master wafer. Following the device fabrication, preparation of both cultivation medium as well as seeding culture is required. Additionally, the microscope hardware and pumping periphery should be set up. Afterwards, the MC device can be loaded with cells and steadily perfused with fresh cultivation medium. The last step consists of adjusting the software settings and starting the live-cell imaging. Subsequent to the successful live-cell imaging, data curation and image analysis follow on the microfluidic cultivation workflow. Yet, comprehensive automated image analysis pipelines are rare. As this sector is not further discussed here, the reader is referred to [44].

### 2.1. Microfluidic Design and Fabrication

For PDMS-based microfluidic devices, a master wafer is required [45]. To fabricate this master wafer, first the microfluidic channel system and cultivation chambers must be designed using CAD software [46]. Several points are important for the correct choice of a promising microfluidic chip design, which are closely related to each other: reliable trapping of the cells in the cultivation unit, sufficient nutrient supply, the possibility of long-term cultivation, and the respective organism’s characteristics. Depending on the research question, the cultivation units are adapted and designed. As an example, for the cultivation of cells in a 2D chamber design, the following points are crucial: The height of channel and chamber layer should have a ratio in a way that fluid flow is restricted to supply channels, consequently mass exchange between channel and chamber only occurs diffusively [47] and possible air bubbles do not affect cultivation chambers substantially. The width and height of the supply channel depends on the cells’ dimensions since the channel must be wide enough to avoid clogging during the loading process. Likewise, the height of the cultivation chamber depends on the applied cells. Cells with a cell wall/stiff membrane can be squeezed into chambers with a height lower than their diameter, while cells with a flexible membrane cannot be retained that way. For motile deformable cells, a cultivation chamber with small entrances or retention structures should be applied. Generally, a larger cultivation chamber can result in the formation of gradients within the cultivation area. Mass exchange and thereby potentially arising gradients can be calculated in advance using computational fluid dynamics (CFD) simulations or can be determined experimentally by trace substance experiments [47,48,49].

Following on designing the microfluidic structures, the master wafer can either be fabricated by photolithographic steps [11,50] applying a photomask, by laser cutting methods or it can be directly fabricated by stereolithography (3D printing) [12].

### 2.2. PDMS Chip Assembly

The next step is the preparation of the microfluidic chip from the master wafer [12,51,52,53,54]. For this purpose, PDMS base and curing agent are mixed in a defined ratio of 10:1 [55]. By changing this ratio, the produced PDMS chip can vary in its stiffness [56], which might also influence cellular behaviour during MC. After proper mixing, the polymer is poured onto the master wafer. As air bubbles are introduced during mixing, the polymer needs to be degassed prior to curing it in an oven. To connect the pumping periphery in a later step, inlet as well as outlet holes are punched using a biopsy puncher and both PDMS chip and glass substrate are cleaned with isopropanol. Finally, the PDMS and glass substrate are covalently bonded to each other by applying oxygen-plasma for surface activation [57,58]. A final baking step for bonding strength can be performed at 60 to 90 °C for 30 s to 20 min [54,55,59,60,61].

### 2.3. Cell and Medium Preparation

Following the chip fabrication, the next step for MC is to prepare the biological culture. Regarding biological preparations, the focus lies on the applied medium and the seed train procedure, meaning how many different pre- and intercultures are executed before the microfluidic experiment is performed [45,55]. For microfluidic experiments, the medium is prepared as usual but should be sterile filtrated to remove potentially disturbing particles [62]. If the medium’s pH value is CO_2_-buffered, adjusting the prepared medium to its proper CO_2_-concentration is of utmost importance. For this the prepared medium should be equilibrated to the correct pH value in an CO_2_ incubator. Additionally, gas-tight tubing and pumping periphery is highly recommendable to not risk a change in pH value by CO_2_ degassing. It is beneficial to start the seed train with a cryo-culture of a working cell bank to ensure biological identical starting points. The cryo-culture is used to inoculate a preculture, using the standard cultivation medium. For bacterial experimentation, after sufficient growth in preculture, the main culture can be inoculated with a low cell density [62]. In case of mammalian cells, for reproducibility a consistent number of passages must be maintained prior to the microfluidic experiment [63]. For fungal experiments, cells are plated out on plates and grown for 5–7 days, then harvested and diluted to approximately 10^5^–10^7^ cfu/mL and loaded into the microfluidic device [64]. In the case of algae, the precultures can be inoculated on agarose plates and then transferred to liquid culture where they are cultivated until the start of the experiment (<1 d) [65].

### 2.4. Hardware Preparation

Parallel to the chip fabrication, the live-cell imaging hardware for MC must be set up. To guarantee a stable cultivation environment, potential heating of the microscope incubator needs to be adjusted and the temperature sensor should be placed near the chip. As metal parts do not warm up immediately, heating-up should take place in advance. If necessary for the experiment, CO_2_ atmosphere should be regulated to an appropriate concentration as well.

Considering the live-cell imaging microscope, different configurations have to be checked and readjusted before beginning an experiment. For optimal illumination, the optical path should be centred by Köhler illumination [66,67]. In case of phase contrast microscopy, the phase ring needs to be centred as well to guarantee optimal contrast [67].

After preparation of the periphery and adjusting the microscope, the microfluidic device needs to be placed onto the microscope stage. To prevent the chip from moving during cultivation, a holder should be used, and the chip should be fixed properly by clamping or taping. To maintain image quality and experimental reproducibility, chip mounting needs to be performed thoroughly so that no shift in position or focus occurs.

### 2.5. Device Loading

For the inoculation of cells, the microfluidic device is flushed with cell suspension. In a randomised process, for most of the devices the cultivation chambers are statistically loaded with cells by moving the cell suspension back and forth within the chip, so that pressure differences or air bubbles push the cells into the chambers [68]. With a higher cell density, more chambers are loaded than with a lower cell density. When a sufficient number of chambers are filled with cells, the flow of the cell suspension is stopped. In case of cultivation compartments that are difficult to load with cells, as it is the case with 1D cultivation chambers like mother machines, alternative proceedings like centrifugation may be applied [57]. Depending on the microfluidic design some chips allow cell seeding by special vacuum channels [69,70].

### 2.6. Cultivation

After successfully loading the device with cells, the chip can be connected to the pumping periphery for constant medium supply [55,57]. For this purpose, the loading syringe including tubing is removed from the chip, and the resulting change in pressure has no effect on the trapped cells. The needle should be removed in such a way that a drop of liquid remains on the inlet. Before connecting the periphery to the microfluidic device, the pumps must be calibrated according to the respective setup, so that the desired volume can be pumped precisely. When using syringe pumps, the syringe’s diameter has to be considered for precise pumping, as most software calculate flow rates based on the diameter. Before perfusion can be established, tubing has to be cut and assembled. For the connection between syringe and tubing, special adapters are required. For the use of pressure-driven pumps the medium has to be prepared in sterile medium reservoirs. Additionally, after applying the correct inlet pressure, pumps have to be calibrated. Regardless of the pumping periphery, tubing can be equipped with needles for the connection to the microfluidic device. For some devices, the tubing can also be connected to the microfluidic device without needles. When connecting to the microfluidic device, all tubing should be filled with medium so that no air is introduced into the chip.

Depending on the purpose of the intended cultivation it is helpful to program a specific duration for pumping or setting a target volume. Since there is no feedback from the liquid level inside the syringe or medium reservoir, the target volume or pumping duration must not exceed the prepared medium’s volume.

### 2.7. Live-Cell Imaging

Successful live-cell imaging starts with the implementation of microscopic hardware and software settings [55] and pumping periphery. For the microscope, the most important factors are the choice of objective, the associated phase ring, and the correct setting of the light path, for example directed to the camera. After setting up the microscopic hardware, the software can be adjusted for live-cell imaging. The exposure time and light intensity should be set for optimal image quality. If fluorescence microscopy is requested, the respective filter cubes should be selected, and light intensity and exposure time need to be set independently from light microscopy. The optimal fluorescence intensity and exposure time should be tested and adjusted in advance in the live image to prevent photobleaching during cultivation.

When relevant positions of the microfluidic device are picked, the frequency of imaging has to be selected. The interval between consecutive images depends on the respective growth behaviour of the cultivated cells but is limited by the speed of the microscope stage and the image acquisition speed for the number of marked positions. For long-term cultivations, the focus-drift compensation function or focus system, if available, should be used to avoid focus shift during cultivation.

## 3. Challenges during a MC Experiment

**Figure 3:**
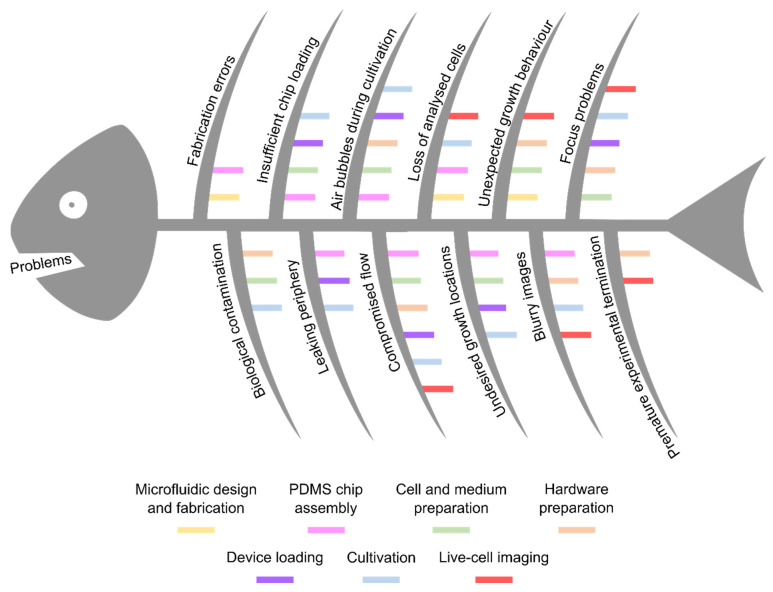
Fishbone diagram of the most frequent challenges during MC. The challenges are arranged according to their temporal order along the workflow of an experiment. Small, coloured fishbones indicate the experimental step in which these challenges most probably have their origin. The introduced colour code matches the colouring of the previous workflow diagram (Figure 2).

Performing MCs is a daily challenge and for most cell types detailed protocols are lacking. In the following a problem-oriented guideline to cope with the most common challenges during MC is presented. The fishbone diagram in Figure 3 gives an overview of the most frequently occurring challenges sorted by their temporal order along the workflow of a MC experiment. The smaller fishbones of each individual challenge indicate the consecutive step of the MC workflow, in which this obstacle might have its origin, to directly identify a starting point for required troubleshooting. Following on the general overview, potential causes for the respective challenges are identified and in-detail solution statements are proposed. Some aspects are additionally illustrated by exemplary images in the Appendix A.

### 3.1. Fabrication Errors

**Figure A1:**
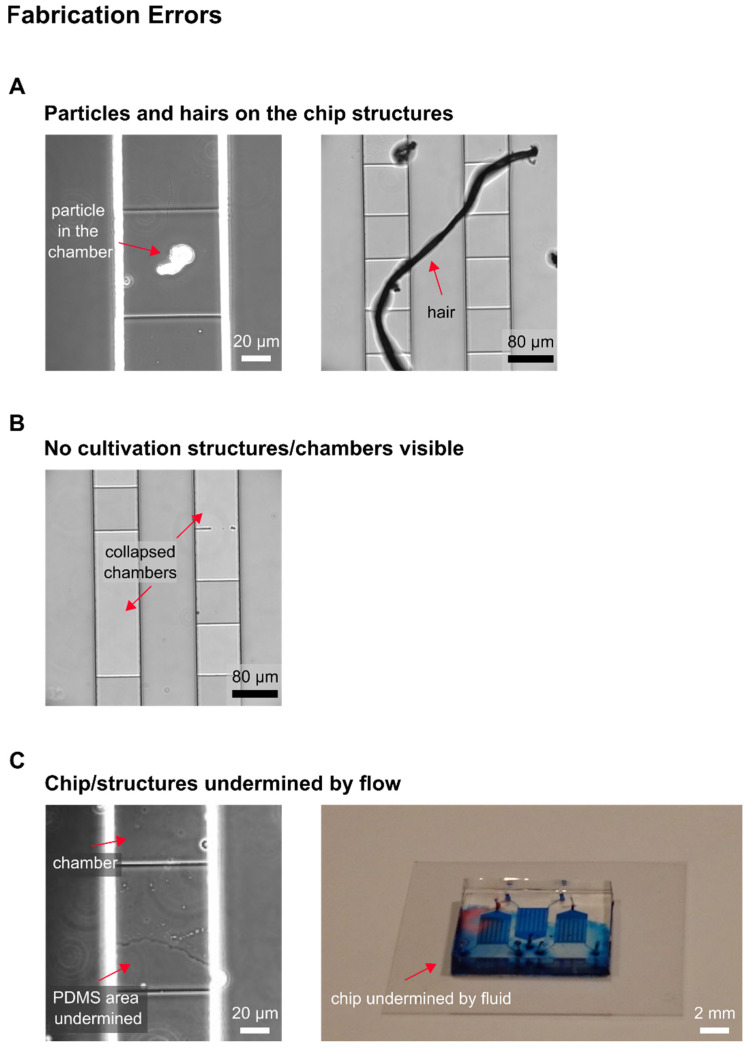
Example images of the fabrication errors (see Table 1). Microscope images of (**A**) Particles and hairs on the chip structures. (**B**) No cultivation structures/chambers visible. (**C**) Chip/structures undermined by fluid.

Every MC experiment starts with the fabrication and assembly of the MC device. Unfortunately, problems that occur during these steps cannot be noticed before the assembled chip is examined under the microscope or the perfusion is started (Table 1). Here, particles or hairs on the microfluidic structures is one of the most frequent disturbances as well as losing the cultivation structures e.g., collapsed chambers, during assembly or structures that are undermined by the fluid flow (Figure A1).

### 3.2. Biological Contamination

In parallel to the assembly of a fully functional MC device, the inoculation culture is prepared. As MC comes with time-consuming preparation, contaminations are very unpleasant but happen at times, as the whole live-cell imaging setup cannot be placed in a sterile environment. Either there is an additional organism growing inside the microfluidic device or only the contaminant made its way into the device and the target organism is not cultivated at all (Table 2). Depending on those two options, the contamination can already be assumed to have happened during the seed train or the contaminant entered the chip during an ongoing MC.

### 3.3. Inefficient Chip Loading

**Figure A2:**
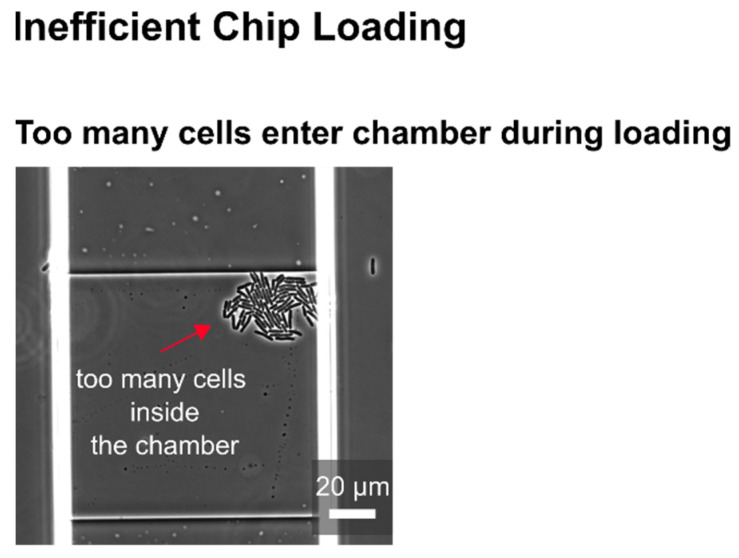
Example images of inefficient chip loading (see Table 3). Microscope images of too many cells enter chamber during loading.

Depending on the purpose of the MC, it is desirable to load the device either with few cells if single-cell analyses are planned or with multiple cells in one cultivation chamber for e.g., cell interaction studies. Hence, loading too many or too few cells into the microfluidic device can compromise the experiment before the MC even began (Table 3). Most frequently these problems are connected to the inoculation culture’s cell density but can also have their origin in air bubbles on-chip or the occurrence of misdirected fluid flow (Figure A2).

### 3.4. Leaking Periphery

**Figure A3:**
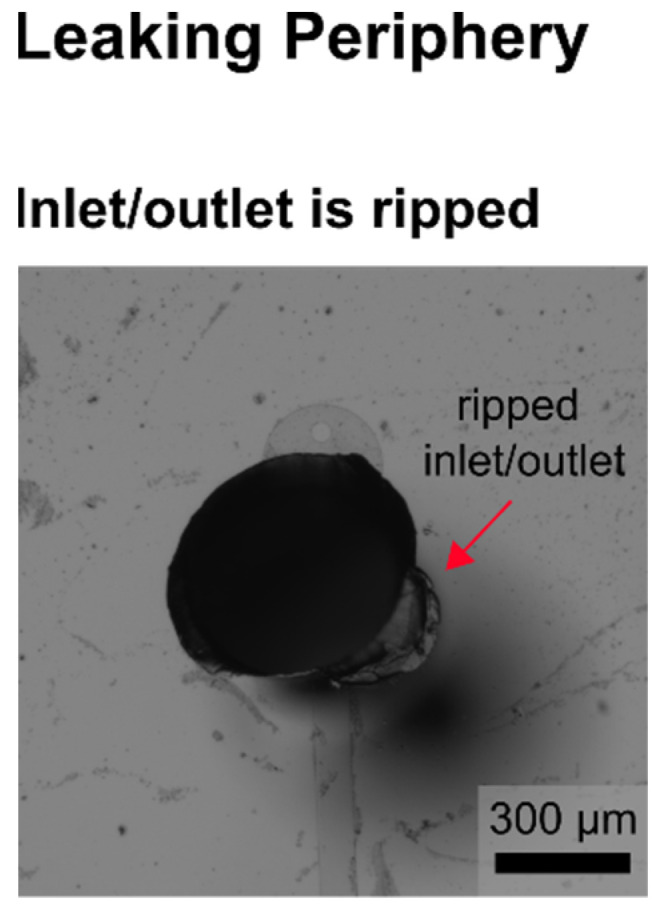
Example images of leaking periphery (see Table 4). Microscope image of Inlet/outlet is ripped.

As a MC is supposed to take place under constant environmental conditions, a leaking periphery automatically implies some aberrations from these conditions. Additionally, leakage represents an entry for contaminations and might also cause damage to essential setup components like the pumping periphery or the live-cell imaging microscope. Basically, leakage can originate from every part of the pumping setup as well as the tubing and connection to the microfluidic device (Table 4). Depending on where the medium drops or puddles are arising, either defective material like damaged adapters or inaccurate preparation like ripped inlet/outlet might be the cause (Figure A3).

### 3.5. Air Bubbles during Cultivation

**Figure A4:**
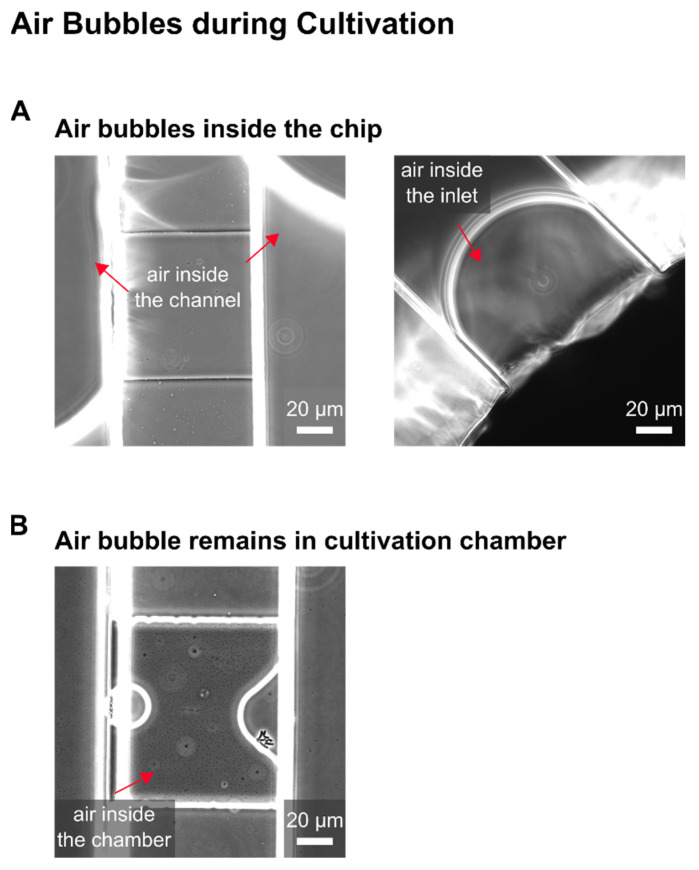
Example images of air bubbles during cultivation (see Table 5). Microscope images of (**A**) Air bubbles inside the chip and (**B**) air bubble remains in cultivation chamber.

If air bubbles occur inside the microfluidic device during the cultivation, they always are a problem by themselves but also cause multiple other anomalies from a controlled cultivation experiment. Air bubbles can sit inside the chip itself (Figure A4) or in the pumping periphery, meaning the syringes, media reservoirs or even the tubing. Most of the time, air is introduced to the cultivation setup by careless handling but in addition it can also form on-chip during a running cultivation experiment (Table 5).

### 3.6. Compromised Flow

**Figure A5:**
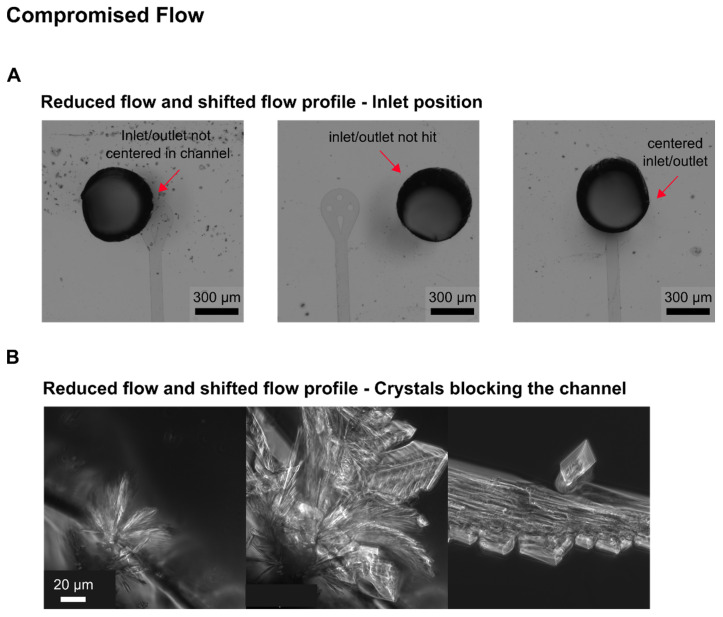
Example images of compromised flow (see Table 6). Microscope images of (**A**) reduced flow and shifted flow profile based on different inlet positions and (**B**) crystals that could block the channel and lead to a reduced flow and shifted flow profile.

Regardless of constant cultivation or periodical switches between different medium conditions, compromised flow characteristics always lead to irreproducible cultivation conditions on-chip and thereby non-reproducible experiments. A disturbance in flow can consist of a reduction in flow rate but also a complete stop. Likewise, the flow direction can be changed so that determined flow profiles do not apply anymore or the flow is directed straight through the cultivation chambers (Table 6). Possible causes for altered flow properties are manifold and range from bad chip design and faulty preparation to defective materials, bad inlet positions (Figure A5) or imprecise software settings.

### 3.7. Loss of Analysed Cells

The loss of cells during cultivation can negatively influence the evaluation. Nevertheless, it can happen that individual cells leave the cultivation chambers or are pushed out during the course of MC, which is not always recorded despite the high temporal resolution using live-cell imaging. Reasons for this can be the chamber height, which could enable the cells to move out of the chamber, or a flow through the chamber caused by air bubbles (Table 7).

### 3.8. Undesired Growth Locations

**Figure A6:**
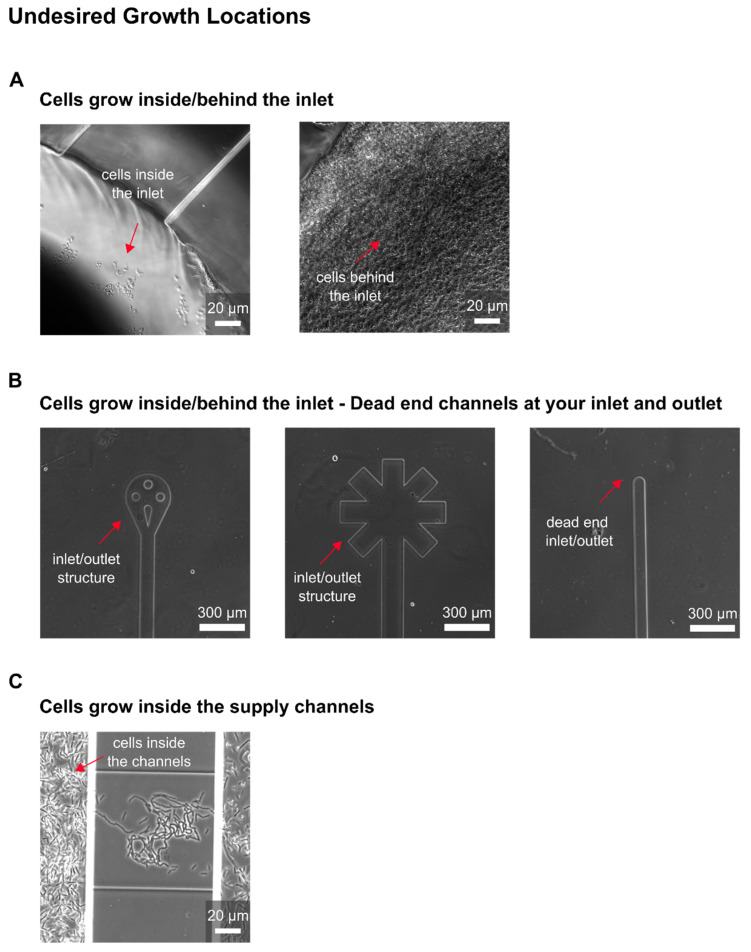
Example images of undesired growth locations (see Table 8). Microscope images of (**A**) cells grow inside/behind the inlet, (**B**) in different inlet structures and (**C**) cells grow inside the supply channels.

A common problem is the growth of cells outside the cultivation chambers. The cells can adhere to the PDMS and grow inside the channel or also at inlets/outlets (Figure A6), which can affect the flow profile up to no flow inside the channel (Table 8). In addition, cells can grow below the PDMS barriers between the chambers, which is caused by partial bonding.

### 3.9. Unexpected Growth Behaviour

During MC, the cells may show different growth behaviour than expected. This can be an increase of division time/decrease of growth rate, growth arrest after a few cell divisions, changes in cell morphology or even no cell division during cultivation. The causes for these observations can be numerous e.g., low viability of the inoculation culture, too much stress during cultivation or too high light exposure parameters during cultivation (Table 9). A detailed analysis of this challenge can be found in Section 4.

### 3.10. Blurry Images

**Figure A7:**
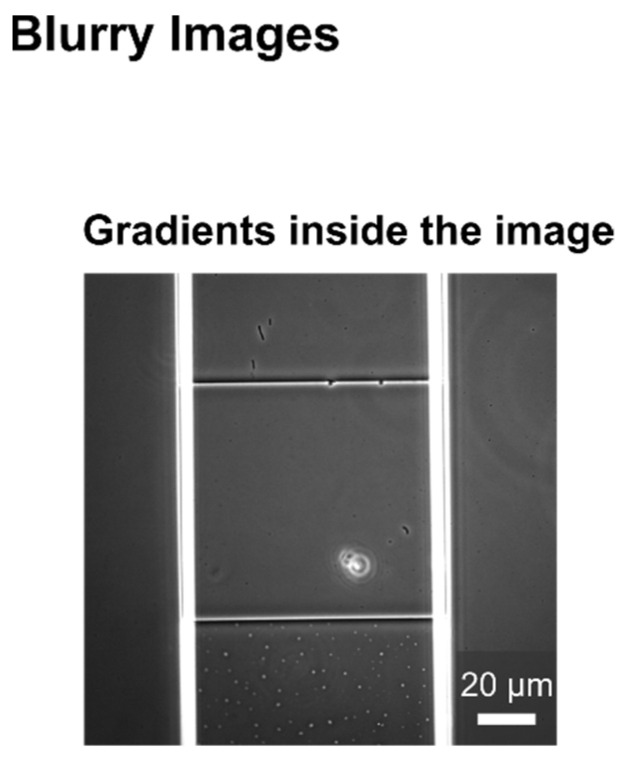
Example images of blurry images (see Table 10). Microscope image of gradients inside the image.

A crucial hurdle during MC is the quality of the live-cell images. A common problem that reduces quality are gradients within the images (Figure A7) that make subsequent analysis of cell number or morphology in an automated manner difficult or impossible. These gradients can be caused by e.g., different brightness within the image, only partial image illumination or colour/grey gradients caused by light scattering (Table 10). Another problem are shadows in the microscope image, caused for example by hardware like tubing or needles in the light path. The time invested in correcting the microscopic settings at the beginning of each experiment will therefore significantly improve the quality of the image data and save time during subsequent analysis.

### 3.11. Focus Problems

**Figure A8:**
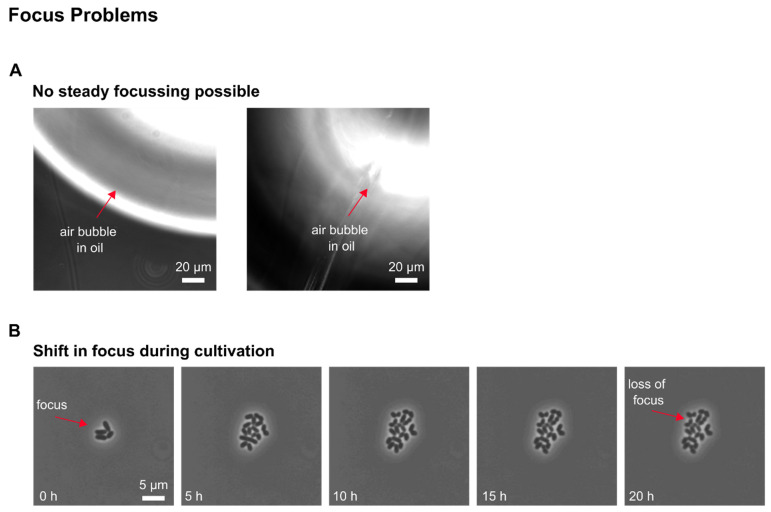
Example images of focus problems (see Table 11). Microscope images of (**A**) no steady focussing possible based on a bubble in the immersion oil and (**B**) shift in focus during cultivation.

The focus of the live-cell images during cultivation is crucial for later data analysis and interpretation. Cell counting can be automated to a certain extent with suitable software. The software recognises and separates cells based on differences in grey value. If these are subjected to gradients, automated image analysis becomes increasingly unreliable. Cellular morphology, and thus the single cell data, is also difficult to analyse by software if the acquired image is out of focus. The focus problems can be caused by e.g., air bubbles in the chip, a permanent loss of focus during cultivation due to air bubbles on the oil film, or a focus shift during cultivation due to an unfirmly installed chip (Table 11, Figure A8).

### 3.12. Premature Experimental Termination

The final and unpredictable hurdle during MC is the occurrence of software or hardware problems. In this case, the biological and technical parts of the experiment function perfectly. Yet the cultivation or live-cell imaging might stop earlier than originally planned. This is usually due to faulty software settings (Table 12). Another problem is that the live-cell imaging data cannot be saved because the hard disk space is exhausted. In addition, a shutdown of the computer or even of the entire experimental setup could happen due to power failures.

## 4. Case Studies

Setting up MC experiments can be hard work. Before desired experiments and analysis can be performed, establishing reliable growth of the desirable organism inside a MC device represents one of the first obstacles. Not only in microfluidics but also in other experimental scales it is well known that during developing novel cultivation systems, cells might not grow at first, since cultivation conditions are not adequately met by the new device [73,74]. After the identification of beneficial growth conditions, guaranteeing reproducibility is the next critical step towards systematic and quantitative studies. However, not only establishing growth but also performing negative-control experiments can be particularly demanding in microfluidic systems.

**Figure 4:**
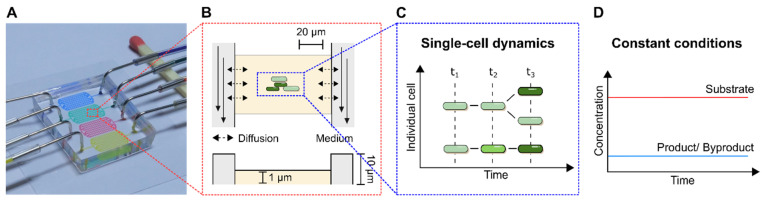
Microfluidic single-cell cultivation (MSCC) and its characteristics. (**A**) Microfluidic PDMS-glass cultivation device. (**B**) Schematic figure of a 2D cultivation chamber with its dimensions and characteristic flow profile. (**C**) Analysis of single-cell dynamics investigating growth-related and morphological heterogeneities. (**D**) Nutrient profile inside a 2D cultivation chamber during MSCC.

In the following, selected examples of these challenges in microfluidic single-cell cultivation (MSCC) approaches (Figure 4A) are presented and suitable troubleshooting strategies are proposed in detail. As a subclass of MC, MSCC allows the cultivation of cells with single-cell resolution and thereby the investigation of single-cell dynamics or population heterogeneities with high spatio-temporal resolution. For this purpose, most commonly 2D cultivation chamber designs are utilised (Figure 4B), that result in monolayer growth of the applied organisms [14]. Due to the resulting spatial restriction, cultivated cells stay in one focal plane during live-cell imaging, which allows time resolved analysis of single-cell behaviour in contrast to population average measurements (Figure 4C). Additionally, cultivation volumes range from nanolitre to picolitre scale, resulting in constant nutrient supply and defined cultivation conditions over the whole cultivation time (Figure 4D).

### 4.1. Case Study I—Establishing MSCC for a New Organism

It is not unusual that cells, when exposed to a different cultivation environment, need time for adaption, which in most cases results in a distinct lag-phase. Likewise, only a fraction of cells might restart growth when discrepancies between the former and the new cultivation environment are given [75]. Therefore, this behaviour especially occurs when cultivation protocols of well-established organisms in lab-scale are transferred to MSCC. With minimising the cultivation volume in microfluidic devices, formerly dispensable physical properties like surface tension, surface-to-volume ratio, laminar flow, and diffusive mass exchange become crucial. At the same time, running MSCC under constant perfusion holds unique challenges ready, that do not arise in other cultivation scales and operating modes like the constant wash-out of secreted (by-)products and a constant oversupply of nutrients (Figure 4D) [14]. Recently, we developed a novel MSCC platform for the long-term cultivation of Chinese hamster ovary (CHO) suspension cells [63] and therefore had to overcome several of these challenges.

The development of our MSCC device resulted in successful trapping of CHO cells. After optimizing the loading procedure regarding the cell density and loading strategy, as mammalian cell lines are known to be compromised in physiology by increasing shear stress, in first instance, we obtained cells that did not grow; only residual morphological changes and occasional division events were observable. As performed in shake flask or bioreactor cultivations, our MSCC experiment was set up at a cultivation temperature of 37 °C with steady perfusion of commercially available chemically defined, serum-free medium (TCX6D, Xell AG, Bielefeld, Germany). Since air bubbles constantly entered the system during MSCC, we assumed compromised cultivation conditions regarding nutrient supply to be the reason for the absence of steady cellular growth. To identify whether air was pumped into the chip or arose gradually inside the channels, observing the devices inlets during live-cell imaging proved helpful. Yet, most of the time air bubbles were introduced during connecting the pumping periphery, which made special precautions necessary like connecting needles to wetted inlets or removing stuck air bubbles from the inlet mechanically. Additionally, we increased flow rate from 0.8 µL/min to 2 µL/min to shorten residence time of the perfused medium inside our device and lowered the supply channel’s height. Thereby, we reduced the device’s total volume to compensate evaporation of liquid through the PDMS chip. With a 10-times medium exchange per minute throughout the whole device, we successfully minimised gas formation on-chip.

After adapting the microfluidic design concerning supply channel and chamber height as well as the cultivation protocol, the first MSCC runs again failed after a few days, as CHO cells still did not divide properly inside our device. When vital, the cells show constantly occurring protuberances of their cellular membrane during live-cell imaging, which arise from steadily secretion of vesicles [63]. Here, we observed a persistent change of cellular morphology to a smooth surface followed by cell death after a few more days. Since cell death did not occur immediately after loading the cells and still sporadic cell division events were observable, we assumed its origin to be not connected to preculture handling but to appear in a later stage of cultivation. To check whether PDMS, which is often described as harmful for cell culture [76], might cause cell death, we washed the PDMS chips with n-Pentane to remove uncrosslinked monomers, but cells did not grow anyway. Therefore, we ruled out PDMS as reason and focused on other components of our setup e.g., the tubing. For bacterial MSCC, Tygon^®^ is a common material utilised for tubing. Although its status as biocompatible, there have been reports [77] of negative influence on cell culture due to leachables. To control if these leachables have a negative effect on CHO cells, we exchanged the previously applied Tygon^®^ tubing against PTFE tubing. Another reason for exchanging Tygon^®^ against PTFE tubing was the lower gas permeability of PTFE: Many cell culture media are CO_2_ buffered, thus losing the adjusted CO_2_ concentration might lead to a pH shift and thereby to non-optimal growth conditions. Using an additional CO_2_ incubation chamber for long-term MSCC also helped to guarantee stable CO_2_ atmosphere in the chip’s surrounding.

Changing the tubing of the device led to cellular survival during MSCC but growth still stopped after 24 to 48 h and cells stayed in an unchanged status till the end of cultivation. Since cells did not demise, we concluded the problem not to be a technical one but rather assumed some limitations that prevented CHO cells from ongoing cell division. Looking at single-cell cloning procedures, growing isolated cells is also highly challenging when chemically defined media without any serum-percentage are applied. In MSCC all secreted factors were additionally diluted and washed out directly due to the constant perfusion of the device, which prohibited the accumulation of hormones and other beneficial factors. Without any single-cell medium available in the market, we decided to mix fresh medium with already conditioned medium from the main culture’s exponential growth phase to supply the isolated cells with essential signalling molecules and thus mislead them over their solitude inside our microfluidic device. This had already been successfully tested for various organisms such as bacteria [78,79]. With a ratio of 1:1, cells finally continued growing until the whole cultivation chamber was filled. However, generating the conditioned medium from the exponential growth phase seemed to be of utmost importance, as medium from the late stationary phase did not promote single-cell growth comparably but rather retarded it. For a more detailed explanation of the results of this case study, the reader is referred to Schmitz et al. [63].

The realisation of MSCC for an organism not previously cultivated in microfluidic devices is associated with varying challenges which are individual for every new organism. For CHO suspension cells the unknown environment and materials turned out to be not only problematic but even toxic. Therefore, significant improvements could be made by adapting the tubing and guaranteeing a constant CO_2_ atmosphere during MSCC. Finally, supplementing perfusion medium with conditioned medium lead to stable growth. Initial non-growing behaviour of cells in minimal medium commonly can be tackled by introducing complex components into the medium. However, some complex compounds might alter cellular behaviour drastically like serum might trigger adherent growth in cell culture, compromising proper analysis. As a result, defined and reproducible cultivation cannot be performed.

### 4.2. Case Study II—Getting Cells to Grow Reproducibly

After MSCC setup and cultivation protocol have been successfully established for a new organism, getting the cells to grow in a reproducible way for quantitative single-cell studies still can be difficult to achieve. This can only be accomplished by using defined minimal medium, as a complex medium can slightly vary in composition and thus the cells’ behaviour might be affected concerning morphology or growth rate up to a complete growth arrest [80]. However, for quantitative measurements it is important that the cells grow reproducibly to answer different research questions. Therefore, it must be investigated whether these irregularities have a technical or a biological origin. In most cases, a variety of parameters can affect the reproducibility of cellular growth. In the following, we present some cases that show which reasons may underlie non-reproducible growth.

**Figure 5:**
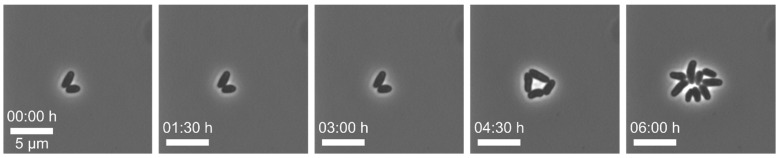
Microscopic images of *C. glutamicum* with an increased lag phase in the first three hours of cultivation (scale bar = 5 µm).

Example 1—increased lag phase of cells: In this series of experiments, *Corynebacterium glutamicum* was cultivated under constant CGXII minimal medium [27,81] conditions at 30 °C in an open-box monolayer cultivation chamber (2D chamber) [82]. After cell loading of our MSCC chip, we observed a distinct lag phase of all cells (see Figure 5). As a first step, we examined the pre- and interculture and found that the growth phase of the main culture was a crucial factor for the initial lag phase on-chip. An increased lag-phase in the MSCC was observed when cells from the late exponential phase were applied for chip inoculation. We found that in precultures of an optical density (OD_600_) above 0.5 the PCA is depleted from the medium and consequently the cells’ metabolism is changed [27]. To prevent this, precultures were inoculated with an OD_600_ between 0.05 and 0.1 and applied for chip loading when the OD_600_ reached values between 0.2 and 0.4. We inoculated the MSCC device with an early exponential growth phase (OD_600_~0.3) which resulted in reproducible growth of *C. glutamicum* cells with no observable lag-phase.

**Figure 6:**
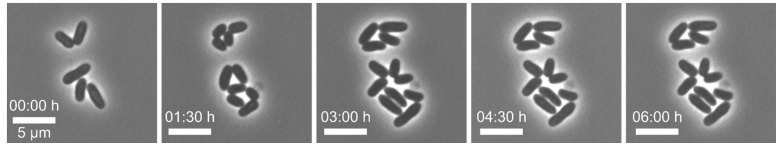
Microscopic images of *C. glutamicum* with stagnating growth after three hours of cultivation (scale bar = 5 µm).

Example 2—stagnating growth of cells: To study the growth under optimal conditions, we cultivated *C. glutamicum* in a 2D cultivation chamber system with constant perfusion of CGXII minimal medium at 30 °C. The minimal medium CGXII consists of six components: a base solution, CaCl_2_, biotin, glucose, protocatechuic acid (PCA) and a trace elements solution. During this MSCC experiment, we observed just a few cell divisions (see Figure 6), mostly two to three divisions at the beginning of the experiment, until growth completely stagnated. After excluding the microfluidic setup and its periphery as an error source, we checked the pre- and interculture to see if there were any problems during the shake flask cultivation that may affect the subsequent MSCC experiment. We found that the 3-(N-morpholino) propane sulfonic acid (MOPS) buffer in our CGXII medium was omitted during shake flask cultivation, since buffering medium in MSCC is not as relevant as in batch cultivations, due to the constant perfusion. As a result, there was a pH shift because of the lack of buffering during pre-cultivation seed train. After preparing the CGXII medium with MOPS buffer for shake flask cultivation, we unexpectedly still noticed decreased growth and finally growth arrest during MSCC experiments. Thus, we switched from the minimal medium CGXII to the complex medium BHI for MSCC and observed an optimal growth rate (µ ≈ 0.9 h^−1^) [62]. Based on the obtained results, we concluded that the decreased growth rate during the experiments was related to the minimal medium CGXII and its components. Various media components such as the base solution and glucose were systematically replaced with new stock solutions, but normal growth in CGXII medium during MSCC was not restored. Subsequently two more components of the applied medium were checked: the iron chelator PCA and the inserted biotin solution. We tested different stock solutions and found that using a different iron chelator such as citrate resulted in exponential growth for *C. glutamicum* in MSCC. Therefore, the cause of our problem was a compromised PCA solution. A new PCA stock solution finally resulted in regular growth on-chip (µ ≈ 0.6 h^−1^) [82]. For a more detail explanation of the results of this case study, the reader is referred to Grünberger et al. [22] and Täuber et al. [62].

**Figure 7:**
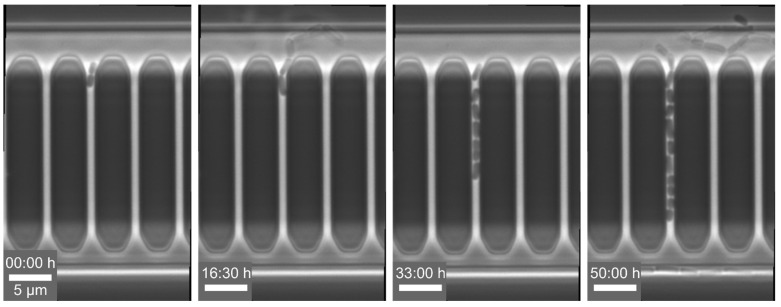
Microscopic images of *C. glutamicum* showing a prolonged division time of the cells due to the mechanical stress caused by the only 0.8 µm wide cultivation channels (scale bar = 5 µm).

Example 3—prolonged division time of cells: For the analysis of single-cell events, we cultivated *C. glutamicum* with constant CGXII medium supply at 30 °C in a 1D cultivation chamber design, modified mother machines, with a length of 20 µm and a width of 0.8 µm, which is open to the supply channel on both sides. Here, we observed an increase in doubling time (see Figure 7) compared to cells which were cultivated in 2D cultivation chamber systems. Typically, *C. glutamicum* divides in a V-shape, but under spatial restrictions, the cells no longer form this V-shape and divide along their main axis. The additional mechanical stress during division increased the doubling time. Similar observations have been reported by Yang et al. [83] and Dusny et al. [84] when cells are cultivated in agarose-pads. Therefore, we developed wider 1D cultivation chambers with a width between 1.5 and 2 µm, in which the cells finally can divide in their preferred V-shape again. With the adapted cultivation chamber design, we observed V-shaped division of our cells as well as optimal doubling times of t_D_ = 70 min.

**Figure 8:**
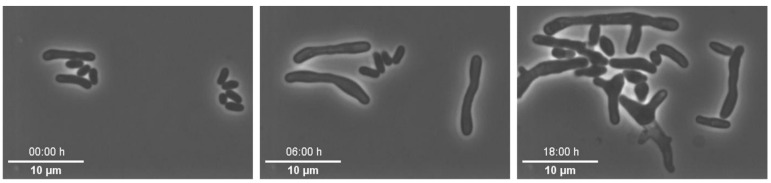
Microscopic images of *C. glutamicum* in which the morphology of the cells was already different at the beginning of cultivation and had changed further in the course of cultivation (scale bar = 10 µm).

Example 4—altered morphology of cells: In this set of experiments, we cultivated *C. glutamicum* under constant CGXII medium supply, but with varying pH values at 30 °C to investigate its pH stress response [82]. For each experiment, pH was adjusted to the desired pH value between pH 6 and 8. During all of these experiments we observed altered cellular morphology and non-uniform cell growth (see Figure 8), which was not expectable based on previous experiments. First, we tested the individual medium components of the CGXII medium to determine if they were the reason for the inconsistent growth behaviour, but normal growth could not be restored. As we cultivated *C. glutamicum* in the CGXII medium without pH adjustment, optimal growth was restored. Based on this result, we suspected that the cause of the altered morphology and growth rates must be related to the pH adjustment procedure. Therefore, we tested different approaches to exclude impurities. We prepared medium and adjusted the pH using different pH meters to rule out instrumental failure as a source of error but altered growth morphology remained unchanged. Afterwards, the pipettes used to transfer the pH adjusting agent into the medium were examined in more detail. Different pipettes were used: plastic single-use pipette tips, plastic Pasteur pipettes and glass Pasteur pipettes. After adjusting the pH value with plastic Pasteur pipettes and single-use tips, reduced growth and altered morphology still were observed. However, utilizing glass Pasteur pipettes resulted in expected growth and morphology. Thus, we suspect that leachable substances were released from the plastic pipettes as well as single-use pipettes. Consequently, we decided to make future pH adjustments with glass pipettes only. For a more detailed explanation of the results of this case study, the reader is referred to Täuber et al. [82].

**Figure 9:**
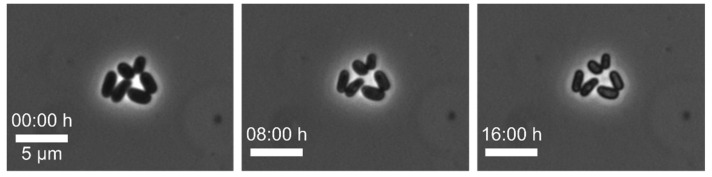
Microscopic images of *C. glutamicum* where no growth is seen in the chip over the whole cultivation time (scale bar = 5 µm).

Example 5—no growth on-chip: In this experiment, we cultivated *C. glutamicum* with BHI (complex) medium supply at 30 °C in perfusion to analyse the colony growth rate. However, no growth was observed in the MSCC device (see Figure 9). To check whether the origin of the absence of growth was medium related, we cultivated our cells in CGXII minimal medium but were not able to detect any growth as well. Therefore, we assumed that this problem had its origin in a technical step prior to the MSCC experiment. We suspected the cleaning procedure of our PDMS chips and glass slides as critical step. We usually clean both parts, PDMS chip and glass slide, with isopropanol, thus it could be possible that isopropanol residues remained in the channel after cleaning, which will be toxic to the cells and most likely affect the growth. Therefore, we adapted our cleaning procedure in two different ways. First, we tried to wash the PDMS chip with ultrapure water after cleaning it with isopropanol to remove potential isopropanol residues. Second, we stored the PDMS chip overnight after cleaning with isopropanol before bonding the PDMS chip and the glass slide, which allowed the isopropanol residue to evaporate from the PDMS chip. With the new cleaning procedure, the cells grew optimally in MSCC experiments, therefore, both methods turned out to be successful in removing isopropanol residues.

**Figure 10:**
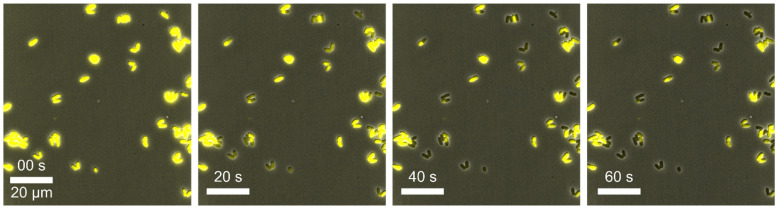
Microscopic images of *C. glutamicum* DM1800 pSenLysTK in which cell death of cells occurs by photobleaching at a fluorescence intensity of 100% and exposure time of 100 ms (scale bar = 20 µm).

Example 6—cell death: In this experiment, *C. glutamicum* DM1800 pSenLysTK [85] was cultivated in CGXII medium at 30 °C under constant conditions in 2D cultivation chambers. This strain on the one hand produces intracellular L-lysine, which, on the other hand, triggers the production of a yellow fluorescent protein (YFP) under the control of the lysine sensor pSenLys [85]. This YFP molecule can be examined by fluorescence microscopy so that lysine production behaviour is quantifiable. During the MSCC experiments, we observed a steadily decrease in the fluorescence signal over the cultivation time (see Figure 10) as well as a decrease in growth resulting finally in cell death for most of the analysed cells. We assumed that the decrease in fluorescence signal could be related to photobleaching. In this process, the fluorophore can decay, releasing harmful products like reactive oxygen species and hence lead to cell death [86]. Based on this assumption, we investigated the fluorescence settings in more detail. We performed several cultivations with different fluorescence settings for the excitation light intensity and exposure time and found optimal parameters for our bacterial strain. The settings of the excitation intensities highly depend on the sensitivity and stability of the fluorophore in use as well as the sensitivity of the organisms. These parameters can be easily adjusted in live-cell imaging, where photobleaching directly represents the response to the corresponding excitation intensity. For our strain, an exposure time in the lower millisecond range and a low light intensity of less than 10% of the maximum intensity proved to be optimal [87]. Using the correct fluorescence settings, no impairments of the fluorescence signal or growth was observed. 

To sum up, achieving reproducible growth during MSCC is a very challenging task, as every single step, from microfluidic design over biological preparation to the microscope settings has to be considered as a potential source of influence on cellular growth and morphology. Based on our experience, the reason for altered growth often originates before the experimental step of live-cell imaging and the MSCC. Neglecting the importance of a proper seed train and properly stored chemicals or media already hampers the cultivation before it even started. Likewise, careless chip cleaning or adjusting the medium’s pH value with single-use plastic pipettes can show an effect which unfortunately is not noticeable until growth rates are determined after MSCC. During the actual experiment, cellular growth might be unexpectedly altered by spatial restriction or too intense light exposure. Because of the variety of sources of error, troubleshooting must start again from the beginning for each challenge that arises and does not follow a pre-set order, as it was the case in Case study I, and workflows must be followed in detail.

### 4.3. Case Study III—Growing Cells without Carbon Source

Performing successful MSCC experiments include the execution of negative control experiments, as for any study in microbiology and applied biotechnology. These experiments are necessary to validate well-designed scientific experiments and findings. As described before, quantitative growth studies rely on the use of minimal and defined medium allowing to draw conclusive interpretation on the obtained MSCC data. After the selection and design of the desired medium, one typical control experiment is the cultivation of cells without the main carbon source. In the past, these experiments revealed significant scientific surprises and led to the development of adjusted microfluidic cultivation media. Here, we will demonstrate the importance on the example of *C. glutamicum* WT as well as *Escherichia coli* MG1655 K12.

*C. glutamicum* MSCC experiments are typically performed with the well-established CGXII minimal medium (see Case study II). Analysing microcolony growth with multiple replicates repeatably resulted in growth rates around µ ≈ 0.15 h^−1,^ when glucose was omitted within the CGXII medium (negative control experiments). Thus, we concluded that the microbial cells must gain their demanded carbon from a yet unknown source [27]. In successive steps, PDMS chips were washed with n-Pentane to remove any monomer residuals that were in suspicion to be metabolised by the organism. In the next step, tubing was exchanged to rule out leakage of carbon-based material into the experimental setup. After exclusion of these factors, we were convinced that the residual growth must be based on a carbon source that had to be part of the medium composition. After a careful review of the CGXII compounds, only MOPS as a buffer compound and PCA as iron chelator have been identified as compounds that contain carbon elements. Since pH conditions are constant in perfusion cultivations, MOPS was omitted from the cultivation medium. The experiments still revealed a remaining growth rate of approximately µ ≈ 0.15 h^−1^ and thus we could conclude that MOPS was not responsible for residual growth. Removing the iron chelator PCA from the cultivation medium finally resulted in zero growth [27]. However, simply removing the iron chelator for further limitation studies to analyse carbon source limitation was not a solution, since its primary function cannot be compensated by other medium ingredients. On the first instance, negative control experiments were successful. Unfortunately, *C. glutamicum* was not able to grow on a main carbon source (here glucose) when PCA was omitted due to its function as iron chelator. After repetitive experimental trial and error, we identified citrate as an alternative iron chelator, that was not metabolised as carbon source at CGXII medium composition at the given environmental conditions (unpublished data).

In a similar study, *E. coli* cells were cultivated under limiting condition in M9 medium [88]. Here, cellular growth was determined at different carbon source concentrations ranging from pM to mM. Under limiting conditions, decreased growth rates up to the point of zero growth were expected. Again, at limiting concentrations and without any carbon source, significant cellular growth was detectable (unpublished data). Biased from our previous experience with *C. glutamicum*, our first approach was to check whether the applied minimal medium exhibits any potential secondary carbon sources like the iron chelator ethylenediaminetetraacetic acid (EDTA). Any experiments altering the iron chelator molecule and its concentration resulted in residual growth at zero and limiting carbon conditions, thus we concluded that not the medium compounds per se lead to the remaining growth within MSCC. In the next step, we logically checked all base chemicals for manufacturer related contaminants that could be metabolised by the cells. Here, no notable elements have been found. Next, we examined the containers and bottles in which the various stock solutions were prepared and stored. The preparation of new stock solutions resulted in the expected non-growth at low carbon conditions. In the following, we separated workflows in cleaning procedures and the preparation of stock solution and medium preparation of medium containing no or only small amounts of carbon source. As a result, quantitative reproducible experiments at low carbon concentrations were obtained.

Using growth medium with its standard component concentrations for MSCC of just a few cells always results in excessive supply of nutrients. Therefore, even reducing e.g., carbon source inside the medium drastically will still allow full-speed growth of the loaded cells, although cells cultivated in bigger scales might reach carbon limitations already after a few hours of cultivation. Operating MSCC in perfusion mode makes it even more difficult to achieve real limiting conditions. As we had to find out, even medium components like PCA, that are not sufficient to promote steady growth in shake flasks or bioreactors because of their low concentration, enable constant growth in a MSCC. Likewise, minimal contaminations of the applied bottles during medium preparation might result in decreased but still detectable growth. Thus, not only the choice of medium is crucial but also its preparation for MSCC has to be even more careful than for conventional cultivation approaches.

## 5. Conclusions

All in all, performing successful and quantitative MC experiments relies on a proper design selection, preculture and careful choice of the cultivation medium. Likewise, the subsequent handling of the experimental periphery, loading of the MC device with cells, and the live-cell imaging process have to be executed with reasonable care. Cultivation in microscale can be seen as magnifying glasses: Important details regarding different growth characteristics can be identified, at the same time overlaying effects, that are typically not detectable in bulk scale, cannot be ignored or can have a significant influence on every microfluidic experiment, as shown on selected examples for MSCC.

Although there are only few commercially available setups, more and more easy to use custom made MC devices are being developed to enable application without excessive microfluidic training. However, when these tools are applied by untrained end-users, multiple challenges may occur during MC along the overall workflow from chip fabrication to final on-chip cultivation, making experiments and interpretation unfeasible. Several approaches to solve these problems are obvious, while more inconclusive challenges might have their origin in multiple steps so that troubleshooting becomes hardly performable for beginners. Here, for the first time, a generalised guideline is provided to troubleshoot the most common challenges for setting up a successful MC experiment. As every single step in MC can be topic of its own review article, the most important procedures and critical factors of the entire workflow have been succinctly summarised here. Subsequently, the reader is provided with a compendium of possible causes for the most prominent challenges with respective solution statements in form of a checklist. With the additional case studies, examining the problems of establishing MSCC for a new organism, performing reproducible MSCC experiments, and preforming negative-control experiments, examples of how the provided lists can be applied for troubleshooting are given.

We are convinced that our guideline will prove exquisitely handy for every untrained user of MC devices. This may help to broaden the field of application for microfluidic analysis and cultivation and thereby increase its prominence aside from already established MC applications.

## Figures and Tables

**Table 1:** Listed are the most frequently occurring challenges concerning fabrication errors with their respective cause and a suggested solution.

Fabrication Errors		
Problem	Cause	Solution
Particles and hairs on the chip structures	Non-particle free working environment	Work under a flow bench/in a clean room and regularly clean the surfaces used.
		Use adhesive tape to clean the chip’s structures. Note: At low chamber height, this might damage the chambers.
		Clean the plasma equipment regularly.
		Intensive clean: use an ultrasonic bath for chip cleaning. For example, with acetone, ethanol, and water for 30 s each.
No cultivation structures/chambers visible	Collapsed chambers	Do not press on the bonded chip.
		For small structures: assemble carefully, glass slide on top of PDMS chip.
		Increase the time between the activation and bonding.
		Reduce the PDMS layer thickness.
	Structures on the wafer are destroyed	Create a new master wafer.
Chip/structures undermined by fluid	Partial bonding	Use a scalpel or tweezers to carefully press on the chip, but never on the structures, to enhance bonding.
		Use a final baking step at 60–90 °C for 1 to 20 min.
	No bonding	Check the oxygen concentration, pressure, and vacuum inside the plasma chamber.
		Increase activation time.
		Check for grease residues on the chip and repeat cleaning procedure.
		Check if no other materials e.g., metal, are in the plasma chamber.
		When placing the PDMS chip and glass slide on each other, avoid bending the chip.
Failed PDMS moulding	PDMS sticks tightly to the master mould after moulding	Silanise the master mould before the first replication.

**Table 2:** Listed are the most frequently occurring challenges concerning biological contaminations with their respective cause and a suggested solution.

Biological Contamination		
Problem	Cause	Solution
Additional organism grows inside the device	Contamination after chip loading	Use gloves during assembly of the periphery.
		Rinse tubing and the chip thoroughly with medium after connecting all periphery parts.
		Check after the connection that no medium and liquid is on top of the chip to prevent contaminants from entering the chip this way.
		Fill waste tube with disinfectant.
	Contamination in syringe/medium reservoir	Check medium for contaminations.
		Use sterile single-use syringes or medium reservoirs.
		Autoclave the adapters. Afterwards, check if adapter sealing (glue) is still intact.
		Autoclave the tubing, if it complies with the manufacturer’s data sheet.
		Connect the adapter and tubing in a clean environment (clean bench).
Cells show different morphology or physiology than expected	Contamination during seed train	Work under a clean bench.
		Use sterile equipment.
		Autoclave or sterile filtrate media components.
		Check that there are no contaminants in your cryo-culture, check cryovials for contamination (before freezing or use).
		Check medium for contaminations.
		Check preculture for contaminations.

**Table 3:** Listed are the most frequently occurring challenges concerning inefficient chip loading with their respective cause and a suggested solution.

Inefficient Chip Loading		
Problem	Cause	Solution
No cells enter chamber during loading	Remaining air inside chambers prevents seeding	Start loading process directly after chip bonding before surfaces became hydrophobic again.
	Low cell density in main culture	Use air bubbles for loading.
		Check if your cells are sedimented at the syringe’s wall. If so, the cells can be resuspended by carefully shaking the syringe.
		Increase cultivation time of main culture.
		Use higher starting cell density in main culture.
		Concentrate cell suspension by e.g., centrifugation.
Too many cells enter chamber during loading	High cell density in main culture	Use a syringe with medium to wash cells out of the chambers.
		Check the growth phase of your cells.
		Decrease cultivation time of main culture.
		Dilute cell suspension to appropriate cell density.
		Use lower starting cell density for your main culture.
	Flow through the chambers caused by particles/hair accumulates cells	See “Fabrication Errors“ and “Compromised Flow”.
	Cell aggregates within device	Solve aggregates by mechanical force or chemicals.
	Concentrated cell density in lower part of the loading syringe	Fill the cells into a vessel and let it shake just until inoculation.
		Use the density medium to adjust the carrying medium for more uniformed suspension.

**Table 4:** Listed are the most frequently occurring challenges concerning leaking periphery with their respective cause and a suggested solution.

Leaking Periphery		
Problem	Cause	Solution
Medium drop on top of the inlet/outlet	Inlet/outlet ripped	After punching, carefully remove the puncher with rotating motions.
		Use a sharp puncher.
		Punch on soft ground e.g., PDMS, so that the puncher is not damaged.
	Leakage at needle	Use a puncher with a smaller diameter than the needle.
		Check that the inlet is straight.
		Check that the needle does not stick too deep in the inlet.
	PDMS residue of punching left in inlet/outlet	Ensure that the residues are removed after punching.
Medium drops on pumping periphery	Leakage at syringe piston	Do not clamp the syringe too tightly to prevent it from deformation.
		Check that the syringe is in one plane with the substrate/underground.
		Check that the plunger is seated correctly.
		Use glass syringes. They cannot be deformed.
	Leakage between syringe and adapter	Check if the adapter sits tightly on the syringe’s nose.
		Check if the adhesive bond between adapter and needle is intact.
		Use Luer-lock syringes.
Medium flow between glass slide and PDMS chip	Chip not fully bonded	See “Fabrication Errors”.

**Table 5:** Listed are the most frequently occurring challenges concerning air bubbles during cultivation with their respective cause and a suggested solution.

Air Bubbles during Cultivation		
Problem	Cause	Solution
Air bubbles inside the chip	Air remained inside the channel after loading	Remove air by flushing the chip thoroughly with medium after loading.
	Air remained inside the inlet after connecting the pumping periphery	Connect wetted needle with moistened inlet.
		Flush the chip with medium from the outlet. If air does not vanish, use a needle or syringe adapter to remove air mechanically from the inlet.
		Syringe pumps: connect tubing/needle with the chip before mounting the syringe.
		Pressure pumps: shortly increase the pressure to press air through PDMS.
Air bubbles inside the pumping periphery	Air inside the syringe adapter	Hold the syringe upright with the adapter at the head while flushing the tubing.
		Fill adapter before connecting the wetted syringe.
		Check if the connection between adapter and syringe is tight/not leaky.
	Air bubbles inside the syringe/medium reservoir	Pressure pumps: Centrifuge medium inside the reservoirs before use and do not shake it afterwards.
		Pressure pumps: hang the tubing deeper into the vessel.
		Syringe pumps: fill syringe very slowly to avoid bubble formation.
	Air inside the tubing	Check that your tubing is filled with liquid without bubbles.
		Check for tight/none-leaky connection between adapter and tubing.
	Air bubbles arise inside the medium reservoir/syringe during the cultivation	Prewarm the device and settings at cultivation temperature.
		Keep the room temperature steady to not influence gas solubility.
		Immerse the entire degassed MC chips in sterile water in the middle of cultivation.
Air bubble remains in cultivation chamber	Material turned hydrophobic again	Bond the chip immediately before the experimental start.
	Volume of cultivation chamber is too big	Fill chip with medium and apply vacuum e.g., by a desiccator to remove the remaining air through the PDMS.
		Use a revision of the device with real-time air removal functions or surface treatment [71,72].

**Table 6:** Listed are the most frequently occurring challenges concerning compromised flow with their respective cause and a suggested solution.

Compromised Flow		
Problem	Cause	Solution
Reduced flow and shifted flow profile	Cells grow in the inlets	Use a medium syringe and flush the channels and inlets.
	Leakage	See “Leaking Periphery”.
	Cells in the supply channel	Increase the flow to flush them away.
		If cells adhere, try coating of PDMS using BSA [57] or Pluronic before loading.
	Inlet position	Practice precise punching. Inlet holes should be at the same position at the respective inlet channel.
	Chip design prone to fluctuations	Use buffer zones or broader channels.
	Crystals blocking the channel	Check your solutions for crystals.
		Filtrate your medium before use.
		Check your media/salt composition if pH shift causes proteins to agglomerate or minerals to crystalize.
		Prepare new medium/solutions.
	Altered viscosity of your medium	Before using an unknown medium, test its flow behaviour on-chip and adapt the pressure settings.
One-way flow	Air bubbles block a channel over its complete length	See “Air Bubbles during Cultivation”.
		Use a higher flowrate/pressure settings to flush the bubbles out of the medium.
Flow through the cultivation chamber	Particles within the supply channel	Filtrate medium before use.
		See “Fabrication Errors”.
	Air bubble inside the channel	See “Air Bubbles during Cultivation”.
No fluid flow inside the device	Syringe is leaking	See “Leaking Periphery”.
	Medium reservoir for pressure pumps not closed tightly	Check if the fitting is in the right position and cap is tightly sealed.
	No medium left to pump	Calculate the needed amount for your cultivation time correctly.
	Technical problems	Pressure pumps: check the external pressure.
	Software problems	Check the set volume, flow rate and pumping time of the pumping equipment.
		Check the software that no defined volume or end of flow has been set.

**Table 7:** Listed are the most frequently occurring challenges concerning loss of analysed cells with their respective cause and a suggested solution.

Loss of Analysed Cells		
Problem	Cause	Solution
Cells leave the cultivation chamber	Wrong chamber design: Chamber height is higher than the diameter of the cells	Non-deformable cells: chamber height lower than the diameter of the cells. Deformable cells: use retention structures.
	Active cell movement	Adapt the chamber design to smaller entries or blocking structures.
Cells are pushed out of the cultivation chamber	Flow across the chamber	See “Compromised Flow”.
	Bubbles after loading in the chip	See “Air Bubbles during Cultivation”.
	Pressure pulses by insufficient pumps	Use pumps with a finer threading.
		Change your chip design to broader channels.

**Table 8:** Listed are the most frequently occurring challenges concerning undesired growth locations with their respective cause and a suggested solution.

Undesired Growth Locations		
Problem	Cause	Solution
Cells grow inside/behind the inlet	Dead end channels at your inlet and outlet	Punch the inlet and outlet at the outmost end of your structure.
		Use structures that only show blank channels without any cross/star structure at the inlet and outlet.
	Reduced flow inside the device	See “Compromised Flow”.
Cells grow outside the intended chip design/restriction	Chip not complete bonded	See “Fabrication Errors”.
Cells grow inside the supply channels	Cells grow on particles in the channels	See “Fabrication Errors”.
	Cell aggregates inside channel structures	Separate the cells before loading the chip by e.g., vortexing or trypsin treatment.
		Coat PDMS chip using BSA or Pluronic before loading.
	No flow inside the channels	See “Compromised Flow”.

**Table 9:** Listed are the most frequently occurring challenges concerning unexpected growth behaviour with their respective cause and a suggested solution.

Unexpected Growth Behaviour		
Problem	Cause	Solution
Increased division time	No viable pre-/interculture	Check your media composition and cell viability of each working cell bank before use.
		Inoculate the pre-culture with a higher volume of your cryo-culture.
		Use a pre-culture that is not yet in the stationary phase.
		Check if no wrong (incompatible) antibiotics were used.
	Too much stress during loading	Load the chip carefully, do not use too much pressure.
	Wrong temperature	Check that the incubation cage is closed.
		Check the cooling and heating settings.
		Check the location of the temperature sensor, place it near the chip.
		Check the connection between heater and cage whether hoses are bent.
Cells interrupt their growth	Wrong illumination parameters	Decrease light exposure time and intensity.
		Check if the LED is switched off or if the shutter is shut when no image is taken.
	pH shift	Adjust the pH value of the medium, if necessary, by adapting it to a suitable concentration of e.g., CO_2_.
Cells do not divide	Incompatibility with chemostat conditions, missing growth factors	Mix your medium with conditioned medium from the interculture’s exponential growth phase.
		Check if you need a pH buffer system during constant perfusion cultivation.
Changes in morphology	Inadequate chamber height	Higher or wider cultivation chamber area.
	Wrong osmolarity	Check if the medium has the right osmolarity and all components are added.
Loss of adherence	Wrong medium	Check if all media components for adherent growth are present e.g., serum.
	Surface incompatibility	Coat the cultivation device with adherence-enhancing proteins e.g., fibronectin.

**Table 10:** Listed are the most frequently occurring challenges concerning blurry images with their respective cause and a suggested solution.

Blurry Images		
Problem	Cause	Solution
Gradients inside the image	Gradient in brightness	Adjust light path of the microscope by Köhler illumination.
	Only partial image illumination	Open the field diaphragm until the whole picture is illuminated.
	Blurry phase contrast image	Adjust phase ring.
	Colour/grey gradient because of light scattering	Cut chip with more distance to the relevant structures to prevent light refraction of trimmed edge.
Shadow on microscope image	Distracting objects in light path	Remove the liquid on top of the chip.
		Check that there are no tubes between chip and the microscope light path.
		Work in a particle free surrounding during chip preparation to avoid enclosed dirt inside PDMS chips.
		Degas your PDMS completely after mixing PDMS base and curing agent to avoid enclosed air bubbles.
	Distracting circular shapes on your image	Remove air bubbles from the immersion oil film.
		Clean the condenser lens.
		Check for particles in the light path inside the microscope or on the camera.
No live image	No phase contrast/brightfield image	Adjust the graduation of the image’s grey values.
		Check if the intensity of your light source and exposure time is high enough.
		Check if light source is switched on and shutters are open.
		Check if light path is directed to eyepieces or camera.
		Check if the revolving nosepiece is in correct position.
	No fluorescence image	See “No phase contrast/brightfield image”
		Check if correct filter cube is selected.
		Recheck the light source configuration.

**Table 11:** Listed are the most frequently occurring challenges concerning focus problems with their respective cause and a suggested solution.

Focus Problems		
Problem	Cause	Solution
Loss of focus	Air bubbles in chip	See “Air Bubbles during Cultivation”.
		Activate and adjust autofocus system.
	Microscope not heated	Check whether the incubator and thus the stage are preheated.
No steady focussing possible	Air bubbles in immersion oil	See “Blurry Images”.
		Remove air bubbles in immersion oil. Replace chip on lens or remove immersion oil and wet lens again.
		Gently blow on oil film.
		Move microscope stage back and forth until the bubbles have gone over the lens’ edge.
	Insufficient positioning in microscope software	Integrate *z*-axis into positioning.
Shift in focus during cultivation	Chip mounted incorrectly	Check if your chip is in-plane.
		Check that the chip is firmly seated in the slide holder.
	Inaccurate starting focus	Check if the selected *z*-axis and the focus are well adjusted.
		Use the focus-drift compensation or focus system function.
	Shaking/hitting of the ground	The microscope should be placed on a vibration-damped table.

**Table 12:** Listed are the most frequently occurring challenges concerning premature experimental termination with their respective cause and a suggested solution.

Premature Experimental Termination		
Problem	Cause	Solution
Cultivation/live-cell imaging is stopped earlier than intended	Software Setting	Check timers in your live-cell imaging program and pumping software.
Microscope data cannot be saved	Data drive capacity low/no access to the cloud	Export old raw data on time and delete it from data drive to assure sufficient storage capacity.
Computer and setup shut down	Blackouts	Keep informed about your facility’s maintenance schedules.
	Updates of operating system	Suppress automatic system updates for the duration of the experiment.

## Data Availability

This paper is not based on original data.

## References

[1] Bhatt G., Bhattacharya S. (2019). Biosensors on chip: A critical review from an aspect of micro/nanoscales. J. Micromanuf..

[2] Tabeling P. (2010). Introduction to Microfluidics.

[3] Liu Y., Jiang X. (2017). Why microfluidics? Merits and trends in chemical synthesis. Lab Chip.

[4] Beebe D.J., Mensing G.A., Walker G.M. (2002). Physics and Applications of Microfluidics in Biology. Annu. Rev. Biomed. Eng..

[5] Nguyen N.-T., Wereley S.T., Shaegh S.A.M. (2019). Fundamentals and Applications of Microfluidics.

[6] Scott S.M., Ali Z. (2021). Fabrication Methods for Microfluidic Devices: An Overview. Micromachines.

[7] Xia Y., Whitesides G.M. (1998). Soft lithography. Annu. Rev. Mater. Sci..

[8] Ji J., Li M., Chen Z., Wang H., Jiang X., Zhuo K., Liu Y., Yang X., Gu Z., Sang S. (2019). In situ fabrication of organic electrochemical transistors on a microfluidic chip. Nano Res..

[9] Jiang K., Xue C., Arya C., Shao C., George E.O., DeVoe D.L., Raghavan S.R. (2011). A new approach to in-situ “micromanufacturing”: Microfluidic fabrication of magnetic and fluorescent chains using chitosan microparticles as building blocks. Small.

[10] Attia U.M., Marson S., Alcock J.R. (2009). Micro-injection moulding of polymer microfluidic devices. Microfluid. Nanofluid..

[11] Waldbaur A., Rapp H., Länge K., Rapp B.E. (2011). Let there be chip—Towards rapid prototyping of microfluidic devices: One-step manufacturing processes. Anal. Methods.

[12] Gale B., Jafek A., Lambert C., Goenner B., Moghimifam H., Nze U., Kamarapu S. (2018). A Review of Current Methods in Microfluidic Device Fabrication and Future Commercialization Prospects. Inventions.

[13] Raj M.K., Chakraborty S. (2020). PDMS microfluidics: A mini review. J. Appl. Polym. Sci..

[14] Grünberger A., Wiechert W., Kohlheyer D. (2014). Single-cell microfluidics: Opportunity for bioprocess development. Curr. Opin. Biotechnol..

[15] Grünberger A., Paczia N., Probst C., Schendzielorz G., Eggeling L., Noack S., Wiechert W., Kohlheyer D. (2012). A disposable picolitre bioreactor for cultivation and investigation of industrially relevant bacteria on the single cell level. Lab Chip.

[16] Di Carlo D., Wu L.Y., Lee L.P. (2006). Dynamic single cell culture array. Lab Chip.

[17] Banaeiyan A., Ahmadpour D., Adiels C., Goksör M. (2013). Hydrodynamic Cell Trapping for High Throughput Single-Cell Applications. Micromachines.

[18] Wu C., Chen R., Liu Y., Yu Z., Jiang Y., Cheng X. (2017). A planar dielectrophoresis-based chip for high-throughput cell pairing. Lab Chip.

[19] Chiou P.Y., Ohta A.T., Wu M.C. (2005). Massively parallel manipulation of single cells and microparticles using optical images. Nature.

[20] Liu W., Dechev N., Foulds I.G., Burke R., Parameswaran A., Park E.J. (2009). A novel permalloy based magnetic single cell micro array. Lab Chip.

[21] Collins D.J., Morahan B., Garcia-Bustos J., Doerig C., Plebanski M., Neild A. (2015). Two-dimensional single-cell patterning with one cell per well driven by surface acoustic waves. Nat. Commun..

[22] Grünberger A., Probst C., Helfrich S., Nanda A., Stute B., Wiechert W., von Lieres E., Nöh K., Frunzke J., Kohlheyer D. (2015). Spatiotemporal microbial single-cell analysis using a high-throughput microfluidics cultivation platform. Cytom. Part A.

[23] Uphoff S. (2018). Real-time dynamics of mutagenesis reveal the chronology of DNA repair and damage tolerance responses in single cells. Proc. Natl. Acad. Sci. USA.

[24] Wang P., Robert L., Pelletier J., Dang W.L., Taddei F., Wright A., Jun S. (2010). Robust Growth of Escherichia coli. Curr. Biol..

[25] Robert L., Ollion J., Robert J., Song X., Matic I., Elez M. (2018). Mutation dynamics and fitness effects followed in single cells. Science.

[26] Dormeyer M., Lentes S., Ballin P., Wilkens M., Klumpp S., Kohlheyer D., Stannek L., Grünberger A., Commichau F.M. (2018). Visualization of tandem repeat mutagenesis in Bacillus subtilis. DNA Repair.

[27] Unthan S., Grünberger A., van Ooyen J., Gätgens J., Heinrich J., Paczia N., Wiechert W., Kohlheyer D., Noack S. (2014). Beyond growth rate 0.6: What drivesCorynebacterium glutamicumto higher growth rates in defined medium. Biotechnol. Bioeng..

[28] Si F., Le Treut G., Sauls J.T., Vadia S., Levin P.A., Jun S. (2019). Mechanistic Origin of Cell-Size Control and Homeostasis in Bacteria. Curr. Biol..

[29] Dusny C., Schmid A. (2015). Microfluidic single-cell analysis links boundary environments and individual microbial phenotypes. Environ. Microbiol..

[30] Elowitz M.B., Leibler S. (2000). A synthetic oscillatory network of transcriptional regulators. Nature.

[31] Atkinson M.R., Savageau M.A., Myers J.T., Ninfa A.J. (2003). Development of Genetic Circuitry Exhibiting Toggle Switch or Oscillatory Behavior in *Escherichia coli*. Cell.

[32] Ryley J., Pereira-Smith O.M. (2006). Microfluidics device for single cell gene expression analysis inSaccharomyces cerevisiae. Yeast.

[33] Stricker J., Cookson S., Bennett M.R., Mather W.H., Tsimring L.S., Hasty J. (2008). A fast, robust and tunable synthetic gene oscillator. Nature.

[34] Toriello N.M., Douglas E.S., Thaitrong N., Hsiao S.C., Francis M.B., Bertozzi C.R., Mathies R.A. (2008). Integrated microfluidic bioprocessor for single-cell gene expression analysis. Proc. Natl. Acad. Sci. USA.

[35] Mustafi N., Grünberger A., Kohlheyer D., Bott M., Frunzke J. (2012). The development and application of a single-cell biosensor for the detection of l-methionine and branched-chain amino acids. Metab. Eng..

[36] van Vliet S., Co A.D., Winkler A.R., Spriewald S., Stecher B., Ackermann M. (2018). Spatially Correlated Gene Expression in Bacterial Groups: The Role of Lineage History, Spatial Gradients, and Cell-Cell Interactions. Cell Syst..

[37] Rosenthal A.Z., Qi Y., Hormoz S., Park J., Li S.H.-J., Elowitz M.B. (2018). Metabolic interactions between dynamic bacterial subpopulations. eLife.

[38] Martins B.M.C., Locke J.C.W. (2015). Microbial individuality: How single-cell heterogeneity enables population level strategies. Curr. Opin. Microbiol..

[39] Torino S., Corrado B., Iodice M., Coppola G. (2018). PDMS-Based Microfluidic Devices for Cell Culture. Invenions.

[40] Lam J., Marklein R.A., Jimenez-Torres J.A., Beebe D.J., Bauer S.R., Sung K.E. (2017). Adaptation of a Simple Microfluidic Platform for High-Dimensional Quantitative Morphological Analysis of Human Mesenchymal Stromal Cells on Polystyrene-Based Substrates. SLAS Technol..

[41] Kim H.S., Devarenne T.P., Han A. (2015). A high-throughput microfluidic single-cell screening platform capable of selective cell extraction. Lab Chip.

[42] Demming S., Sommer B., Llobera A., Rasch D., Krull R., Büttgenbach S. (2011). Disposable parallel poly(dimethylsiloxane) microbioreactor with integrated readout grid for germination screening of Aspergillus ochraceus. Biomicrofluidics.

[43] Jo M.C., Qin L. (2016). Microfluidic Platforms for Yeast-Based Aging Studies. Small.

[44] Jeckel H., Drescher K. (2021). Advances and opportunities in image analysis of bacterial cells and communities. FEMS Microbiol. Rev..

[45] Huberts D.H.E.W., Janssens G.E., Lee S.S., Vizcarra I.A., Heinemann M. (2013). Continuous high-resolution microscopic observation of replicative aging in budding yeast. J. Vis. Exp..

[46] Li S.-S., Ip C.K.M., Tang M.Y.H., Sy S.K.H., Yung S., Chan T.-M., Yang M., Shum H.C., Wong A.S.T. (2017). Modeling Ovarian Cancer Multicellular Spheroid Behavior in a Dynamic 3D Peritoneal Microdevice. J. Vis. Exp..

[47] Westerwalbesloh C., Grünberger A., Stute B., Weber S., Wiechert W., Kohlheyer D., von Lieres E. (2015). Modeling and CFD simulation of nutrient distribution in picoliter bioreactors for bacterial growth studies on single-cell level. Lab Chip.

[48] Liu J.-S., Zhang Y.-Y., Wang Z., Deng J.-Y., Ye X., Xue R.-Y., Ge D., Xu Z. (2017). Design and Validation of a Microfluidic Chip with Micropillar Arrays for Three-dimensional Cell Culture. Chin. J. Anal. Chem..

[49] Bazaz S.R., Kashaninejad N., Azadi S., Patel K., Asadnia M., Jin D., Warkiani M.E. (2019). Rapid Softlithography Using 3D-Printed Molds. Adv. Mater. Technol..

[50] Lei K.F., Labeed F.H., Fatoyinbo H.O. (2014). Chapter 1. Materials and Fabrication Techniques for Nano- and Microfluidic Devices. Microfluidics in Detection Science.

[51] Christoffersson J., Mandenius C.-F. (2019). Fabrication of a Microfluidic Cell Culture Device Using Photolithographic and Soft Lithographic Techniques. Methods Mol. Biol..

[52] McDonald J.C., Duffy D.C., Anderson J.R., Chiu D.T., Wu H., Schueller O.J.A., Whitesides G.M. (2000). Fabrication of microfluidic systems in poly(dimethylsiloxane). Electrophoresis.

[53] Qin D., Xia Y., Whitesides G.M. (2010). Soft lithography for micro- and nanoscale patterning. Nat. Protoc..

[54] Deshpande S., Dekker C. (2018). On-chip microfluidic production of cell-sized liposomes. Nat. Protoc..

[55] Gruenberger A., Probst C., Heyer A., Wiechert W., Frunzke J., Kohlheyer D. (2013). Microfluidic picoliter bioreactor for microbial single-cell analysis: Fabrication, system setup, and operation. J. Vis. Exp..

[56] Jin C., Ma C., Yang Z., Lin H. (2020). A Force Measurement Method Based on Flexible PDMS Grating. Appl. Sci..

[57] Cabeen M.T., Losick R. (2018). Single-cell Microfluidic Analysis of Bacillus subtilis. J. Vis. Exp..

[58] Xiong L., Chen P., Zhou Q. (2014). Adhesion promotion between PDMS and glass by oxygen plasma pre-treatment. J. Adhes. Sci. Technol..

[59] Mazutis L., Gilbert J., Ung W.L., Weitz D.A., Griffiths A.D., Heyman J.A. (2013). Single-cell analysis and sorting using droplet-based microfluidics. Nat. Protoc..

[60] Kim S.H., Yang Y., Kim M., Nam S.-W., Lee K.-M., Lee N.Y., Kim Y.S., Park S. (2007). Simple Route to Hydrophilic Microfluidic Chip Fabrication Using an Ultraviolet (UV)-Cured Polymer. Adv. Funct. Mater..

[61] Bounab Y., Eyer K., Dixneuf S., Rybczynska M., Chauvel C., Mistretta M., Tran T., Aymerich N., Chenon G., Llitjos J.-F. (2020). Dynamic single-cell phenotyping of immune cells using the microfluidic platform DropMap. Nat. Protoc..

[62] Täuber S., Golze C., Ho P., von Lieres E., Grünberger A. (2020). dMSCC: A microfluidic platform for microbial single-cell cultivation of Corynebacterium glutamicum under dynamic environmental medium conditions. Lab Chip.

[63] Schmitz J., Täuber S., Westerwalbesloh C., von Lieres E., Noll T., Grünberger A. (2021). Development and application of a cultivation platform for mammalian suspension cell lines with single-cell resolution. Biotechnol. Bioeng..

[64] Hopke A., Mela A., Ellett F., Carter-House D., Peña J.F., Stajich J.E., Altamirano S., Lovett B., Egan M., Kale S. (2021). Crowdsourced analysis of fungal growth and branching on microfluidic platforms. PLoS ONE.

[65] Westerwalbesloh C., Brehl C., Weber S., Probst C., Widzgowski J., Grünberger A., Pfaff C., Nedbal L., Kohlheyer D. (2019). A microfluidic photobioreactor for simultaneous observation and cultivation of single microalgal cells or cell aggregates. PLoS ONE.

[66] Neuendorf J., Neuendorf J. (2020). Setting-Up Köhler Illumination. Urine Sediment.

[67] Hard R., Hipp J., Tangrea M., Tomaszewski J., McManus L.M., Mitchell R.N. (2014). Applications of Image Science in Pathology and Cell Biology. Pathobiology of Human Disease.

[68] Probst C., Grünberger A., Braun N., Helfrich S., Nöh K., Wiechert W., Kohlheyer D. (2015). Rapid inoculation of single bacteria into parallel picoliter fermentation chambers. Anal. Methods.

[69] Gao Y., Li P., Pappas D. (2013). A microfluidic localized, multiple cell culture array using vacuum actuated cell seeding: Integrated anticancer drug testing. Biomed. Microdevices.

[70] Kolnik M., Tsimring L.S., Hasty J. (2012). Vacuum-assisted cell loading enables shear-free mammalian microfluidic culture. Lab Chip.

[71] Huang C., Wippold J.A., Stratis-Cullum D., Han A. (2020). Eliminating air bubble in microfluidic systems utilizing integrated in-line sloped microstructures. Biomed. Microdevices.

[72] Zheng W., Wang Z., Zhang W., Jiang X. (2010). A simple PDMS-based microfluidic channel design that removes bubbles for long-term on-chip culture of mammalian cells. Lab Chip.

[73] Zengler K., Toledo G., Rappé M., Elkins J., Mathur E.J., Short J.M., Keller M. (2002). Cultivating the uncultured. Proc. Natl. Acad. Sci. USA.

[74] Solden L., Lloyd K., Wrighton K. (2016). The bright side of microbial dark matter: Lessons learned from the uncultivated majority. Curr. Opin. Microbiol..

[75] Kotte O., Volkmer B., Radzikowski J.L., Heinemann M. (2014). Phenotypic bistability in Escherichia coli ’s central carbon metabolism. Mol. Syst. Biol..

[76] Iliescu C., Taylor H., Avram M., Miao J., Franssila S. (2012). A practical guide for the fabrication of microfluidic devices using glass and silicon. Biomicrofluidics.

[77] Cooksey G.A., Elliott J.T., Plant A.L. (2011). Reproducibility and robustness of a real-time microfluidic cell toxicity assay. Anal. Chem..

[78] Dantur K.I., Pizarro R.A. (2004). Effect of growth phase on the Escherichia coli response to ultraviolet-A radiation: Influence of conditioned media, hydrogen peroxide and acetate. J. Photochem. Photobiol. B Biol..

[79] Weichart D.H., Kell D.B. (2001). Characterization of an autostimulatory substance produced by Escherichia coli. Microbiology.

[80] Binder D., Probst C., Grünberger A., Hilgers F., Loeschcke A., Jaeger K.-E., Kohlheyer D., Drepper T. (2016). Comparative Single-Cell Analysis of Different E. coli Expression Systems during Microfluidic Cultivation. PLoS ONE.

[81] Keilhauer C., Eggeling L., Sahm H. (1993). Isoleucine synthesis in Corynebacterium glutamicum: Molecular analysis of the ilvB-ilvN-ilvC operon. J. Bacteriol..

[82] Täuber S., Blöbaum L., Wendisch V.F., Grünberger A. (2021). Growth Response and Recovery of Corynebacterium glutamicum Colonies on Single-Cell Level Upon Defined pH Stress Pulses. Front. Microbiol..

[83] Yang D., Jennings A.D., Borrego E., Retterer S.T., Männik J. (2018). Analysis of Factors Limiting Bacterial Growth in PDMS Mother Machine Devices. Front. Microbiol..

[84] Dusny C., Grünberger A., Probst C., Wiechert W., Kohlheyer D., Schmid A. (2015). Technical bias of microcultivation environments on single-cell physiology. Lab Chip.

[85] Binder S., Schendzielorz G., Stäbler N., Krumbach K., Hoffmann K., Bott M., Eggeling L. (2012). A high-throughput approach to identify genomic variants of bacterial metabolite producers at the single-cell level. Genome Biol..

[86] Duanghathaipornsuk S., Farrell E.J., Alba-Rubio A.C., Zelenay P., Kim D.-S. (2021). Detection Technologies for Reactive Oxygen Species: Fluorescence and Electrochemical Methods and Their Applications. Biosensors.

[87] El Najjar N., Van Teeseling M.C.F., Mayer B., Hermann S., Thanbichler M., Graumann P.L. (2020). Bacterial cell growth is arrested by violet and blue, but not yellow light excitation during fluorescence microscopy. BMC Mol. Cell Biol..

[88] Kangwa M., Yelemane V., Polat A.N., Gorrepati K.D.D., Grasselli M., Fernández-Lahore M. (2015). High-level fed-batch fermentative expression of an engineered Staphylococcal protein A based ligand in E. coli: Purification and characterization. AMB Express.

